# Effects of the Times on the Profession

**Published:** 1842-09

**Authors:** 


*Effects of the Times on the Profession.*—Strange as it may appear to some, those who are most familiar with the professional interests of the country know very well that the existing hard times affect even the profession of medicine. In the first place, fewer books are published, and consequently, one of the avenues, at least, to science, is partially closed. A disposition to curtail individual expenses in order to meet the world as it presents itself, deranged in its affairs, obliges those who always have been the generous patrons of authors and printers, to withhold subscriptions and purchases, and thus this department of industry and labor, connected with medical science, necessarily suffers. New books elicit new ideas in those who read them. Without their multiplication, therefore, there cannot be that mental activity which is produced by the silent, yet cogent stimulus of new treatises, new theories, or the announcement of important discoveries in medicine, surgery and physiology. Thus, in a measure, we are enabled to explain the comparative paucity of original communications in the journals, which are not so generously supplied by correspondents as they have been heretofore. Instead of reading, reflecting and writing as much as formerly, those who were efficient contributors to the scientific periodicals of this country are compelled to bestow much of their attention upon other objects, which the state of the times renders imperative.

In the course of an extensive tour through an enterprising section of the Union, distinguished alike for the fruitfulness of the soil and the vigor and intellectual energy of the inhabitants, the same complaint was heard that has so long rung in our ears at home. And it was a subject of frequent observation, that no class of men were more desponding, or seemed to feel the pressure more severely, than medical practitioners.

3*

Now at first view it may appear absolutely absurd that the scarcity of money, over the civilized world, should so affect the condition of a physician—as though people could not or would not be sick in adverse as well as in prosperous times. The fact is, the hardness of the times has increased the labors of physicians, but they can get little or nothing for their business. Collections, in the country, cannot be made; and the physician, who of all men is dependent on others for the price of his time, finds an increase of fatigue, responsibility and vexation, without obtaining, in many instances, a decent support for his family. Thus, the hardness of the times directly affects the condition of our professional brethren. We are quite sure that this view of the matter is essentially true; and will explain satisfactorily any apparent lack of interest in the advancement of medical science, with those who were formerly distinguished for their activity in promoting its objects and extending its boundaries. The medical practitioner should remember, however, that he may in the end be a loser by retaining the small pecuniary amount which he has been in the habit of paying for medical books and periodicals.—*Boston Med. and Surg. Jour.*

